# Pharmacological Therapies for Bowel Dysfunction After Colorectal Cancer Surgery: A Systematic Review

**DOI:** 10.34172/apb.025.45993

**Published:** 2025-12-23

**Authors:** Alimohammad Bananzadeh, Seyed Matin Emami, Seyed Mohammad Kazem Tadayon, Neda Najib Pour, Sara Shojaei-Zarghani, Seyed Vahid Hosseini

**Affiliations:** ^1^Colorectal Research Center, Shiraz University of Medical Sciences, Shiraz, Iran; ^2^Laparoscopy Research Center, Shiraz University of Medical Sciences, Shiraz, Iran; ^3^School of Medicine, Shiraz University of Medical Sciences, Shiraz, Iran; ^4^Faghihi Hospital, Shiraz University of Medical Sciences, Shiraz, Iran; ^5^Medical Faculty in the Department of Surgery, College of Medicine, University of Saskatchewan, Saskatchewan, Canada

**Keywords:** Colorectal neoplasms, Drug therapy, Fecal incontinence, Low anterior resection syndrome, Systematic review

## Abstract

**Introduction:**

Bowel dysfunction frequently occurs in colorectal cancer (CRC) patients following sphincter-preserving surgeries. This review systematically evaluated the evidence on pharmacological therapies for this complication.

**Methods:**

A systematic search was conducted in PubMed, Web of Science, and Scopus in November 2024. Experimental or quasi-experimental studies examining the effects of conventional pharmacological therapies and herbal medicine on stable CRC patients with post-surgical bowel dysfunction were included.

**Results:**

Among 8,989 retrieved records, eight studies were eligible. These investigated the effects of serotonin type 3 receptor antagonists (n=3), herbal medicines (n=2, specifically Daikenchuto and modified Baizhu Shaoyao San), diazepam (n=1), Botulinum A toxin injection (n=1), and topical phenylephrine (n=1). Except for phenylephrine, interventions showed varying improvements in stool frequency, incontinence, urgency, or quality of life. Most of the included studies exhibited a high risk of bias.

**Conclusion:**

Some interventions appear promising; however, the current evidence is insufficient to guide clinical practice. This review highlights a significant evidence gap and underscores the urgent need for large-scale, rigorous randomized controlled trials to establish definitive therapeutic strategies for this debilitating condition.

## Introduction

 According to Globocan statistics, colorectal cancer (CRC) was the third most commonly diagnosed cancer worldwide, with a five-year prevalence of 5.7 million cases in 2022 (colon cancer: 3,333,832; rectal cancer: 2,274,386).^1^ Surgical resection remains the cornerstone of CRC treatment. Recently, there has been increased interest in sphincter-preserving surgeries as an alternative to methods that result in a permanent stoma for rectal cancer.^2^ However, several patients—particularly those with lower anastomoses, a previous history of temporary protective ileostomy, and those who have undergone adjuvant or neoadjuvant therapy—experience postoperative bowel dysfunction following these surgeries that poses significant management challenges.^3,4^ Common symptoms in these patients include fecal incontinence, painful stools, diarrhea, tenesmus, urgency of stool, and incomplete bowel evacuation, which are typically assessed using the Low Anterior Resection Syndrome (LARS) score.^5^ Furthermore, these patients often experience significant financial strain and a diminished quality of life.^6,7^ Bowel dysfunction is also reported following colon cancer surgeries.^4,8^

 Due to the increasing survival rates of patients following CRC surgeries, there is a growing interest among researchers and clinicians in identifying strategies to enhance their well-being. Several treatment options have been proposed for managing LARS following CRC surgeries. Dietary modifications and pharmacological interventions are considered first-line treatment strategies, followed by additional approaches such as transanal irrigation and neuromodulation.^9^ Access to these advanced therapies is often limited, particularly in low- and middle-income countries. Consequently, pharmacological options can significantly impact patient care, warranting a dedicated review of the current evidence for their efficacy. While previous systematic reviews have examined overall LARS treatment strategies,^10,11^ none have comprehensively focused on available pharmacological therapies. Therefore, this systematic review aimed to synthesize the evidence on pharmacological therapies (both conventional and herbal medicine) for the management of bowel dysfunction following CRC surgeries, assess the quality of the existing studies, identify the knowledge gaps, and to provide direction for future research.

## Methods

###  Search Strategy 

 The present systematic review study was conducted according to the PRISMA 2020 guidelines and the Cochrane Collaboration Handbook for Systematic Reviews of Interventions.^12^ The protocol was registered on PROSPERO (CRD42024607482) and approved by the Ethical Committee of Shiraz University of Medical Sciences (IR.SUMS.MED.REC.1403.635). We conducted a systematic search using Medical Subject Headings (MeSH) terms and keywords identified through expert consultation in PubMed, Web of Science, and Scopus on November 2024. To ensure the identification of all eligible studies, we also searched references and citations of the included studies and Google Scholar. The full search strategy for PubMed is detailed in Supplementary File, Table S1.

###  Study Selection

 Following the database searches, all retrieved records were exported to EndNote 20. After removal of duplicates, titles and abstracts of all studies were screened according to predefined eligibility criteria (Table 1). Studies were excluded at this stage if they did not meet the inclusion criteria based on their titles and abstracts. In cases where there was uncertainty regarding the inclusion or exclusion of a study, the full text was assessed in the subsequent stage. All of these processes were carried out by two independent reviewers, with discrepancies resolved by a third reviewer.

**Table 1:** Eligibility criteria

	**Inclusion**	**Exclusion**
Population	Patients with bowel dysfunction who underwent sphincter-preservation surgeries for sigmoid or rectal cancer (anterior resection, intersphincteric resection, low and very low anterior resection) or sigmoid resection and colectomy for sigmoid and colon cancer Studies on population with heterogeneous malignant and benign conditions (inflammatory bowel diseases, polyps, etc.) were included only if the majority of patients had cancer	Patients with other diseases, Patients with small bowel resections
Intervention	Studies on assessing the effect of all conventional pharmacological therapies (oral, topical, injection, etc.) and herbal medicine (with well-characterized bioactive compounds), after patient stabilization following surgery	Non-pharmacological therapies (nerve stimulation, pelvic floor physiotherapy, etc.) or a combination of pharmacological and non-pharmacological treatments Chemotherapy and immunotherapy drugs
Comparator	Placebo, routine treatment, baseline of the same group	-
Outcome	The primary outcome was bowel function, and the secondary outcomes were quality of life, inflammatory markers, microbiota composition, and anorectal manometry	Bowel function restoration after surgery (first defecation after operation), Effects on side effects of chemoradiation (treatment-related toxicity)
Study design	Experimental or quasi-experimental studies	Review, note, editorial, letter, observational studies, animal studies, protocols

###  Data Extraction

 A pilot form was designed for data extraction. Two reviewers piloted the data-extraction form by independently extracting data from three randomly selected studies. After comparing results and addressing discrepancies, the form was revised; a summary of the extracted data is presented in Table 2.

**Table 2:** Characteristics of the included studies

**First author, year**	**Country**	**Study design**	**Condition**	**Baseline bowel function**	**Analyzed sample size**	**Previous history of CRT**	**Inclusion of IBD patients**	**Men (%)**	**Age (years)**	**Intervention, dose, route of administration**	**Intervention duration**	**Control**	**Type of surgery**	**Time between surgery/stoma closure and start of trial**	**Outcomes**	**Significant findings**
Popeskou SG, 2024^15^	Switzerland	RCT, Cross-over	Rectal cancer	LARS score > 20	38	Yes	NS	45	67.9	Ondansetron, 4 mg/day (b.i.d.), oral	4 weeks in each period (1 week washout)	Placebo	LAR (TME or PME)	> 4 weeks and < 2 years (mean: 8.6 months)	LARS score; incontinence (Vaizey score), quality of life (IBS-QoL)	Improvement of LARS score and incontinence
Sada H, 2023^17^	Japan	RCT, Cross-over	Colon and rectosigmoid cancer	Patients with gastrointestinal symptoms (constipation, difficult defecation, diarrhea, soft stool, feeling of unsatisfied defecation)	20	No	No	100	69.5	Daikenchuto, 15 g/day, oral	28 days in each period (5 days washout)	No treatment	LAR, sigmoidectomy, right-hemicolectomy (25%), Hartmann	> 6 months (median: 28.5 months)	Gastrointestinal symptoms (GSRS, VAS), Sitz-mark transit test, orocecal transit time, Gas Volume Score	Improvement of gastrointestinal symptoms (especially diarrhea and indigestion)
Fei M, 2023^21^	China	RCT	Colon (49%) and rectal (51%) cancer	Recurrent diarrhea	77	No	No	56.2	54	Modified Baizhu Shaoyao San, oral	4 weeks	Loperamide + placebo of Baizhu Shaoyao San	NS	2 weeks	Traditional Chinese medicine syndrome score, plasma motilin and gastrin	Reduction of stool frequency, motilin, and gastrin as well as clinical improvement
Ryoo SB, 2021^19^	South Korea	RCT	Rectal cancer	Stool frequency > 4 times/day, fecal urgency or incontinence	98	Yes	NS	100	60.6	Ramosetron, 5 µg/day, oral	4 weeks	Conservative treatments (Kegel and warm sitz baths)	LAR, ULAR	1 month	LARS score, quality of life (EORTC QLQ-C30), symptom improvement	Reduction of LARS score and bowel frequency; Improvement of global health, emotional and social functioning, appetite, diarrhea; Symptom improvement
Itagaki R, 2014^16^	Japan	Single-arm trial	Rectal cancer (92%) or UC	Uncontrollable urgency or fecal soiling	25	Yes	Yes	100	60	Ramosetron, 5µg/day, oral	4 weeks	No control	LAR, VLAR, ISR, total proctocolectomy with ileal pouch–anastomosis	6 months in 16 cases; 7–12 months in 3 cases; > 1 year in 6 cases	Incontinence (Wexner), urgency grade, number of defecations	Improvement of all outcomes
Bridoux V, 2012^20^	France	Single-arm trial	Rectal cancer (67%) or others	Persistent severe fecal incontinence, high-amplitude contractions, stool leakage	6	Yes	NS	67	57	Intrarectal submucosal injection of 500 U of Botulinum A toxin	Follow up: 6 months after first series of injections; second series of injections for 3 cases within this 6 months	No control	Proctectomy with micro-reservoir coloanal anastomosis (n = 4), STARR (n = 1), implantation of an artificial anal sphincter (n = 1)	8 months to 5 years	Incontinence (Wexner), quality of life (FIQL), anorectal manometry	Improvement of incontinence, FIQL; Reduction of rectal contractile activity
Park JS, 2007^18^	South Korea	RCT	Rectal cancer	Uncontrollable incontinence for at least 6 months after surgery or ileostomy closure	29	Yes	No	62	60.3	30% Phenylephrine (b.i.d.), topically to the anal margin (not intra-anally)	4 weeks	Placebo gel	LAR	> 6 months (duration of symptoms: 15.8 months)	Incontinence (FISI), quality of life (FIQL), anorectal manometry	No statistically significant difference between the two groups
Maeda K, 2002^14^	Japan	Single-arm trial	Rectal cancer	Persistent mild-moderate incontinence	5	No	NS	100	62.5	Diazepam, 2 mg/day, oral	3 months	No control	LAR	At a median of 18 months after surgery and 10 months after stoma closure	Incontinence (Wexner, Miller’s score, Kirwan’s score), anorectal manometry	Improvement of incontinence

AR: anterior resection, b.i.d.: twice daily, CRT: chemoradiotherapy, EORTC QLQ-C30: European Organization for Research and Treatment of Cancer Quality-of-Life Questionnaire Core 30. FIQL: fecal incontinence quality of life, FISI: fecal incontinence severity index, GSRS: gastrointestinal symptom rating scale, IBD: inflammatory bowel disease, IBS-QOL: irritable bowel syndrome quality of life; ISR: intersphincteric resection, LARS: low anterior resection syndrome, NS: not stated, PME: partial mesorectal excision, RCT: randomized controlled trial, STARR: stapled transanal rectal resection; TME: total mesorectal excision, UC: ulcerative colitis, ULAR: ultra-low anterior resection, VAS: visual analogue scale, VLAR: very low anterior resection.

###  Risk of Bias Assessment

 The risk of bias in the included studies was assessed using the Cochrane tool for randomized controlled trials (RCTs) and the ROBINS-I tool for single-arm studies, both evaluated by two independent reviewers. The Cochrane tool considers various types of bias, including selection bias, performance bias, detection bias, attrition bias, selective reporting bias, and other biases related to intervention adherence, using seven questions.^12^ The ROBINS-I tool evaluates bias due to confounding, bias in the selection of participants, bias in the classification of interventions, bias due to deviations from intended interventions, bias due to missing data, bias in the measurement of outcomes, and bias in the selection of reported results.^13^

## Results

###  Study Selection

**Figure 1:**
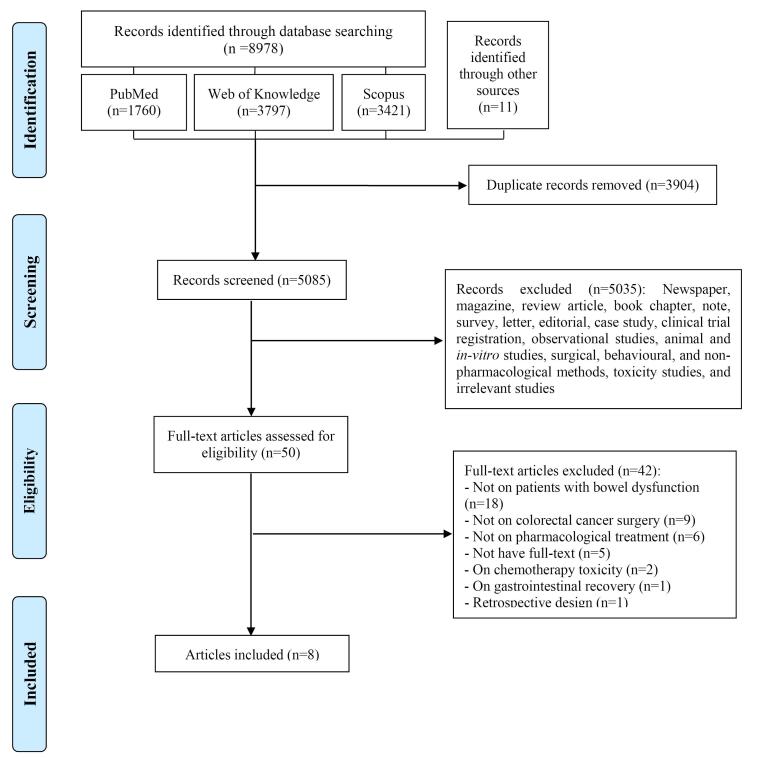
Study Selection Process

 The PRISMA flowchart illustrating the study selection process is presented in Figure 1. The systematic database search yielded 8,978 records. An additional 11 records were identified through Google Scholar and citation tracking, resulting in a total of 8,989 records. After the removal of duplicates, the titles and abstracts of 5,085 studies were screened, resulting in the exclusion of 5,035 studies deemed ineligible. Subsequently, the full texts of the remaining studies were assessed, and ultimately, eight studies were included in our review. The studies excluded at the full-text level are reported in Supplementary File, Table S2.

###  Study Characteristics

 As reported in Table 2, the included studies were conducted between 2002^14^ and 2024.^15^ Among them, three studies were conducted in Japan,^14,16,17^ two in South Korea,^18,19^ and one each in France,^20^ China,^21^ and Switzerland.^15^ Three of the included studies were parallel RCT,^18,19,21^ two were crossover RCT,^15,17^ and three were single-arm trials.^14,16,20^ The control group in the included RCTs received placebo,^15,18^ conservative treatments (Kegel and warm sitz baths),^19^ loperamide,^21^ or no treatment.^17^ In the RCT study by Popeskou *et al*., similar percentages of patients were under loperamide treatment in both groups.^15^ Four of the included studies focused exclusively on men,^14,16,17,19^ while the others included both men and women. The analyzed sample sizes of the included studies ranged from 5^14^ to 98^19^ participants. With the exception of two studies that enrolled a heterogeneous population (including patients who underwent surgery for rectal cancer [ > 67% of participants] or benign diseases)^16,20^, the remaining studies exclusively involved patients with cancer. Two studies addressed colon and rectosigmoid cancers,^17,21^ while the others were on rectal cancer. Studies were on patients with bowel dysfunction at least two weeks post-surgery.^21^ Three studies investigated serotonin (5-hydroxytryptamine [5-HT]) 3 receptor antagonists (ramosetron or ondansetron),^15,16,19^ two evaluated traditional herbal medicines,^17,21^ one assessed diazepam,^14^ one evaluated Botulinum A toxin,^20^ and one examined topical phenylephrine.^18^

 Postoperative bowel function was evaluated using several assessment tools, including the LARS score,^15,19^ Wexner score,^14,16,20^ Vaizey score,^15^ fecal incontinence severity index,^18^ traditional Chinese medicine (TCM) syndrome score,^21^ Miller’s score,^14^ Kirwan’s score,^14^ and/or gastrointestinal symptom rating scale.^17^ Additionally, three studies employed self-developed questionnaires in this regard.^16,19^ Anorectal manometry was assessed in three of the included studies.^14,18,20^ Quality of life also was evaluated in four studies using the European Organization for Research and Treatment of Cancer Quality-of-Life Questionnaire Core 30 (EORTC QLQ-C30),^19^ the irritable bowel syndrome quality of life (IBS-QOL),^15^ and/or fecal incontinence quality of life.^18,20^

###  Risk of Bias Assessment

**Figure 2:**
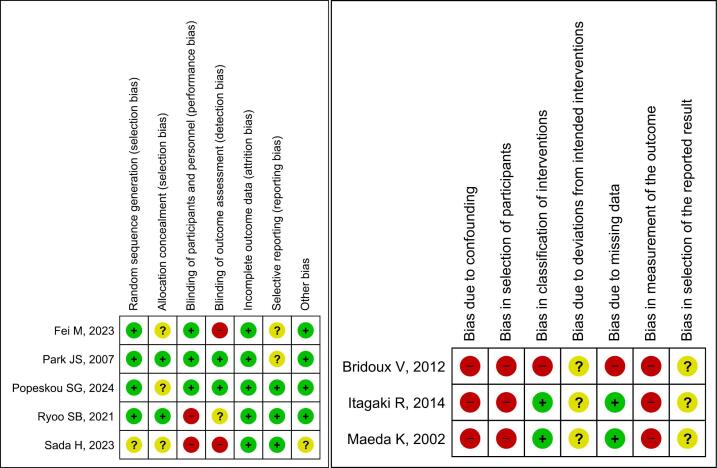
Risk of bias assessment of the included randomized controlled trials (A) and single-arm studies (B)

 The results of the risk of bias assessment for the included RCTs are presented in Figure 2A. Only two of the included studies employed a double-blind design. Furthermore, two studies did not have a registered protocol identifier, and one study lacked clarity regarding the assessment of intervention compliance.

 As depicted in Figure 2B, all included single-arm studies were assessed to be at a critical risk for selection bias and did not adequately account for confounders. In the study by Bridoux *et al*., the intervention was not uniform across all included patients and was determined by the outcomes reported.^20^ Furthermore, there were unaddressed missing data in this study. Although the methods of outcome assessment were comparable across patients in all three studies, the outcomes were subjective and may have been influenced by the non-blinded study design. Additionally, none of the studies had a priori protocols.

###  Outcomes

####  5-HT_3_ Receptor Antagonists

 Serotonin is a neurotransmitter that triggers nausea and vomiting by binding to the 5-HT_3_ receptors located on the gastrointestinal vagal nerve terminals, as well as the chemoreceptor trigger zone in the brain.^22^ The 5-HT_3_ receptor antagonists are US Food and Drug Administration (FDA)-approved antiemetic medications.^22^ Three of the studies included in this review assessed the effects of these agents on patients with bowel dysfunction following CRC surgeries and reported beneficial outcomes. Popeskou *et al*. conducted a crossover RCT to evaluate the effects of ondansetron on patients with minor or major LARS who had undergone surgery between 4 weeks and 2 years prior. They reported improvements in both the LARS score (Cohen’s d in the first period = -0.7, medium effect) and incontinence (Cohen’s d in the first period = -1.5, large effect) among patients treated with ondansetron compared to those receiving a placebo.^15^ However, an incomplete mixed model analysis was conducted in this study.^23^ The effects of administering 5 µg/day of ramosetron for 4 weeks also were investigated in males through one RCT and one single-arm trial involving patients with bowel dysfunction after colorectal surgeries. The findings indicated improvements in the LARS score, as well as reductions in fecal incontinence, urgency, and diarrhea, and enhancements in appetite, global health, and emotional and social functioning.^16,19^ In the RCT by Ryoo *et al*., the effect sizes were large for the LARS score (Cohen’s d = -1.0) and medium for bowel frequency (Cohen’s d = -0.7).

####  Botulinum A Toxin

 Botulinum toxin belongs to the category of neurotoxic medications that possess both therapeutic and cosmetic effects.^24^ One of the studies included in this review evaluated the effects of intrarectal submucosal injection of botulinum toxin type A in six patients suffering from severe fecal incontinence related to overactive rectal contractions; four of these patients had a history of proctectomy for rectal cancer with neoadjuvant chemoradiotherapy, which occurred 8 months to 5 years prior. The injections were administered in a semicircumferential pattern, beginning 1 cm above the coloanal anastomosis, with additional injections performed 5 cm and 10 cm more proximally, resulting in a total dose of 500 units. A second series of injections was conducted within 1 to 6 months following the initial intervention, if improvement was not observed. The study reported significant improvements in fecal incontinence, quality of life, and rectal contractile activity.^20^

####  Phenylephrine

 Phenylephrine is an alpha-1 adrenergic receptor agonist that is approved by the FDA for intravenous use in elevating blood pressure in adults with clinical hypotension. Additionally, its topical formulation is available as an over-the-counter medication for patients with hemorrhoids.^25^ One of the included RCTs evaluated the effects of phenylephrine administered topically to the anal margin in patients experiencing uncontrollable incontinence for at least six months following LAR surgery or ileostomy closure. The study found no significant effects of four weeks of phenylephrine treatment on incontinence, quality of life, or anorectal manometry.^18^

####  Diazepam

 Diazepam is an FDA-approved benzodiazepine medication primarily recognized for its anxiolytic, sedative, and muscle relaxant properties.^26^ The earliest study included in this review evaluated the effects of 2 mg/day of diazepam administered over three months to five patients experiencing persistent mild to moderate incontinence following LAR surgery. The patients included in this study did not exhibit symptoms of anxiety or depression in the enrollment phase or during the trial. In this single-arm study, subjective fecal incontinence symptoms improved significantly from baseline, with a mean reduction of 10.25 units; however, anorectal manometry measurements showed no significant change. Notable improvement began within the first week of the intervention. Furthermore, the recto-anal inhibitory reflex appeared in two cases after the intervention.^14^

####  Traditional Japanese and Chinese Herbal Medicine

 Daikenchuto (DKT) is a traditional Japanese herbal medicine that consists of processed ginger (*Zingiberis Processum Rhizoma*), ginseng (*Panax ginseng*), and Japanese pepper (*Zanthoxylum* fruit).^27^ One of the included studies assessed the effects of 15 g/day of DKT on patients complaining of difficult defecation, diarrhea, soft stools, and a feeling of unsatisfactory defecation following surgery for colon and rectosigmoid cancer. This crossover RCT reported improvements in diarrhea and indigestion.^17^

 In another RCT, Baizhu Shaoyao San, a traditional Chinese herbal formula, was administered to patients with recurrent diarrhea following CRC surgeries. In this study, different modified formulations of Baizhu Shaoyao San were provided based on each patient’s symptoms in addition to diarrhea. The study evaluated the TCM Syndrome Score (which includes stool characteristics, stool frequency, bowel swelling, abdominal pain, abdominal distension, poor appetite, thirst, fatigue, and belching), symptomatic efficacy, and plasma levels of motilin and gastrin. Patients who received Baizhu Shaoyao San demonstrated greater improvements in the TCM Syndrome Score, clinical signs and symptoms of diarrhea, and levels of motilin (Cohen’s d = -0.8) and gastrin (Cohen’s d = -0.7) compared to those who received loperamide (as the control group).^21^

## Discussion

 The increasing survival rate of CRC patients following surgery underscores the need to enhance quality of life. Bowel dysfunction, characterized by symptoms such as fecal incontinence, constipation, diarrhea, and urgency, is a disabling and long-lasting complication that often occurs after CRC surgeries, significantly affecting patients’ well-being.^28,29^ Pharmacological treatments are considered first-line strategies due to their relative affordability and accessibility, particularly in low- and middle-income countries where access to rehabilitative care and advanced surgical techniques is often limited. Furthermore, traditional medicine can serve as culturally acceptable and cost-effective alternatives in these contexts. To date, there are no Food and Drug Administration (FDA)-approved drugs specifically indicated for the treatment of LARS. Antidiarrheal agents, most commonly loperamide, are considered the first-line treatment for LARS; however, their clinical efficacy has primarily been evaluated in the management of diarrhea and fecal incontinence arising from conditions other than CRC resections.^30^

 In this systematic review, we assessed the available literature on the effects of pharmacological treatments for patients suffering from persistent bowel dysfunction after CRC surgeries. Previous studies have investigated the effects of oral 5-HT_3_ receptor antagonists, diazepam, traditional Japanese and Chinese herbal medicines (DKT and modified Baizhu Shaoyao San), as well as botulinum toxin type A injection and topical phenylephrine. With the exception of topical phenylephrine, the other treatments demonstrated promising effects. However, most of the included studies had significant methodological limitations, such as the absence of control groups, lack of blinding, small sample sizes, low statistical power, short intervention durations, and heterogeneous patient populations. Furthermore, due to the limited number of studies for each intervention, conducting a meta-analysis was not feasible. For these reasons, definitive conclusions cannot be drawn. Therefore, despite the promising results observed in the reviewed studies, significant gaps in research remain.

 The improvement of key symptoms associated with LARS, including incontinence, urgency, and increased stool frequency, as well as the resultant emotional and social challenges,^31^ was documented with the use of 5-HT3 receptor antagonists.^15,16,19^ These agents have also been reported to assist in the management of patients with diarrhea-predominant IBS (IBS-D), who experience more frequent defecation, shorter colonic transit times, and urgency^32^ as well as those suffering from diabetic diarrhea.^33^ Several mechanisms may underlie the observed effects of these agents on bowel dysfunction following CRC surgeries. Serotonin has been shown to increase colonic contractions in response to mechanical stretching caused by a stool pellet through the 5-HT3 receptor.^34^ Additionally, stress and anxiety, which are often associated with exacerbated LARS,^35^ have been reported to stimulate colonic transit, motility, and hypersensitivity through the release of serotonin.^36^ These actions can be inhibited by 5-HT3 receptor antagonists.^32^ Furthermore, the inhibitory effects of 5-HT3 receptor antagonists on gut motility persisted even after the depletion of intestinal 5-HT3 receptors, suggesting that these agents may also interact with non-serotonergic pathways.^37^ However, due to the limited availability of high-quality RCTs, further large-scale studies are warranted to elucidate the role of 5-HT3 receptor antagonists on LARS following CRC surgeries and their related mechanisms in this context.

 According to the World Health Organization (WHO), approximately half of the population in several industrialized countries regularly used traditional and complementary medicine in 2012.^38^ Multiple studies assessed the effects of various traditional herbal medicines on CRC and benign gastrointestinal disorders.^39,40^ One included study reported that DKT, a traditional Japanese herbal medicine, improves diarrhea and indigestion following surgery for colon and rectosigmoid cancer.^17^ Furthermore, a previous observational study evaluated the effects of DKT on patients with fecal incontinence or anal sphincter dysfunction. This study reported improvements in fecal incontinence, stool consistency, and maximum resting and squeezing anal pressure.^41^ However, the available data regarding this matter are controversial^42^ and warrant further investigation. The beneficial effects of DKT on fecal incontinence may be related to its impact on the contraction of smooth muscle cells in the internal anal sphincter and the gastrointestinal tract. Additionally, DKT has been reported to modulate gut microbiota,^43^ which could significantly enhance bowel function following CRC surgeries. Besides DKT, ginger and ginseng—two of its ingredients—have shown promising effects in reducing intestinal hypersensitivity and managing IBS-related bowel dysfunction.^44-46^ These findings warrant further investigation in the context of CRC surgeries. Baizhu Shaoyao San—a traditional formula composed of *Atractylodis Macrocephalae Rhizoma*, *Paeoniae Radix Alba*, *Citri Reticulatae Pericarpium*, and *Saposhnikoviae Radix*—has demonstrated efficacy in managing diarrheal conditions, including ulcerative colitis and IBS-D. It also has been shown to significantly reduce stool frequency in patients following CRC resections.^21^ Furthermore, circulating levels of motilin and gastrin, both of which play physiological roles in diarrhea,^47,48^ were also reduced in patients treated with Baizhu Shaoyao San. In addition to traditional herbal medicine, several other studies have evaluated the effects of other complementary and alternative medicine practices, such as acupuncture, on LARS scores following CRC surgeries.^49^ However, the current study did not aim to include these interventions. The literature on this area is currently limited, necessitating further exploration in future research.

 In the present systematic review, we included only studies focusing on patients who suffered from bowel dysfunction following CRC surgeries. However, some other research has investigated the effects of various agents on the prevention of bowel dysfunction after CRC surgeries or on surgeries indicated for reasons other than cancer.^50^ For instance, in the RCT conducted by Park *et al*., multi-strain probiotics were administered to patients undergoing anterior resection for sigmoid colon cancer over a period of four weeks, commencing one week prior to surgery. The probiotics utilized in this study comprised a combination of three strains: *Bifidobacterium animalis* subsp. lactis HY8002, *Lacticaseibacillus casei* HY2782, and *Lactiplantibacillus plantarum* HY7712. The study reported improvements in flatus control and microbiota composition.^51^ Conversely, another study found no significant effect of administering *Lactobacillus plantarum* one day preoperatively and three weeks postoperatively on the incidence of LARS and quality of life.^52^ Given the conflicting evidence, further research is warranted to explore the effects of probiotics on the prevention and management of LARS following CRC surgeries.

 Most drug absorption occurs in the small intestine; therefore, it is unlikely to be significantly altered in the long term by CRC resections. However, due to changes in gastrointestinal anatomy and physiology, altered colonic transit time, shifts in the colonic microbiome, and modifications in hepatic and renal function secondary to surgery-induced physiological stress, the metabolism and efficacy of drugs may be affected.^53^ Currently, there is insufficient data regarding how CRC resections specifically influence the pharmacokinetic properties of the medications discussed in this review or the interactions between drugs and herbs. Future studies are warranted to better elucidate these alterations and to optimize pharmacotherapy in post-surgical CRC patients.

 This study has several limitations. The primary limitation is the high risk of bias in most included studies, largely due to a lack of blinding and the absence of control groups. These methodological shortcomings increase the risk of performance, detection, and selection bias. Furthermore, considerable heterogeneity across the studies precluded a quantitative meta-analysis. Consequently, the findings should be interpreted with caution, and they underscore the necessity for more rigorously designed trials.

## Conclusion

 In conclusion, this systematic review identified preliminary but promising evidence supporting the efficacy of several pharmacological interventions—notably 5-HT3 receptor antagonists, specific herbal formulations, botulinum toxin A, and diazepam—in managing bowel dysfunction following CRC surgery. However, the current evidence is limited by a scarcity of robust studies. Consequently, large-scale, long-term, double-blind, placebo-controlled RCTs are urgently needed to establish therapeutic efficacy and generate robust evidence for clinical practice. Future research should prioritize these agents to validate preliminary findings, clarify mechanisms of action, and explore potential herb–drug interactions. Cost-effectiveness should also be assessed, particularly in low- and middle-income countries where they could represent an accessible first-line strategy.

## Competing Interests

 None.

## Data Availability Statement

 Data are available from the first authors with reasonable request.

## Ethical Approval

 The protocol of this study was approved by the Ethical Committee of Shiraz University of Medical Sciences (IR.SUMS.MED.REC.1403.635).

## Supplementary File

 Table S1. PubMed search strategy
Table S2. Studies excluded in the full-text level
