# Supplementary material for: Pharmacological Therapies for Bowel Dysfunction After Colorectal Cancer Surgery: A Systematic Review
Source: Adv Pharm Bull. 2025 Dec 23;16(1):8–17. doi: 10.34172/apb.025.45993 (PMC13407999; doi:10.34172/apb.025.45993)
Supplement: Supplementary file 1 — Table S1. PubMed search strategy Table S2. Studies excluded in the full-text level [file apb-16-8-s001.pdf]

Adv Pharm Bull 2026;16(1):8-S1
doi:10.34172/apb.025.45993
https://apb.tbzmed.ac.ir

Table S1: PubMed search strategy

**database**	**Search strategy**
MEDLINE (Pubmed)<br>n=1759	(resection [Title/abstract] OR excision [Title/abstract] OR proctectomy [Title/abstract] OR colectomy[Title/abstract]) AND (anterior resection syndrome [Title/abstract] OR bowel dysfunction [Title/abstract] OR fecal incontinence [Title/abstract] OR anal incontinence [Title/abstract] OR bowel function [Title/abstract] OR Bowel Incontinence [Title/abstract] OR Fecal Soiling [Title/abstract] OR Soilings, Fecal [Title/abstract] OR Incontinence, Fecal [Title/abstract] OR Incontinence, Bowel [Title/abstract]) AND (colon*[Title/abstract] OR rect*[Title/abstract] OR colorect*[Title/abstract])

Table S2. Studies excluded in the full-text level

Title	Reason
A comparative study of loperamide and diphenoxylate in the treatment of chronic diarrhoea caused by intestinal resection	Most patients had not cancer
A comparison of the effect of loperamide in oral or suppository form vs placebo in patients with ileo-anal pouches	Not on colorectal cancer surgery
A double-blind randomised controlled clinical trial of Shenling Baizhu Granules in treating low anterior resection syndrome in rectal cancer.	No access to full-text
A Four-Probiotics Regimen Reduces Postoperative Complications After Colorectal Surgery: A Randomized, Double-Blind, Placebo-Controlled Study	Not on patients with bowel dysfunction
Antibiotic treatment for anterior resection syndrome improves patient's subjective bowel function after surgery for rectal cancer: a randomized trial	No access to full-text
Anti-inflammatory effect of the kampo Japanese traditional medicine daikenchuto after colorectal resection	Not on patients with bowel dysfunction
A randomized double-blind placebo-controlled trial of probiotics in post-surgical colorectal cancer	Not on patients with bowel dysfunction
A randomized trial comparing transanal irrigation and percutaneous tibial nerve stimulation in the management of low anterior resection syndrome	Not on pharmacological treatments
Baseline characteristics and recruitment for SWOG S1820: altering intake, managing bowel symptoms in survivors of rectal cancer (AIMS-RC)	Not on pharmacological treatments
Clinical efficacy of buzhong yiqi pill combined with imodium in treating post-operational diarrhea in patients of colonic cancer	No access to full-text
Clinical efficacy of Daikenchuto for gastrointestinal dysfunction following colon surgery: a randomized, double-blind, multicenter, placebo-controlled study (JFMC39-0902)	Not on patients with bowel dysfunction
Clinical trial assessing VSL#3 for the treatment of anterior resection syndrome	Not on patients with bowel dysfunction
Double-blind placebo-controlled study of loperamide (Imodium) in chronic diarrhoea caused by ileocolic disease or resection	Not on colorectal cancer surgery
Early postoperative administration of probiotics versus placebo in elderly patients undergoing elective colorectal surgery: a double-blind randomized controlled trial	Not on patients with bowel dysfunction
Effectiveness and tolerability of colesevelam hydrochloride for bile-acid malabsorption in patients with cancer: a retrospective chart review and patient questionnaire	Not on colorectal cancer surgery
Effect of prucalopride to improve time to gut function recovery following elective colorectal surgery: randomized clinical trial	On gastrointestinal recovery after surgery
Effects of 12 weeks of probiotic supplementation on quality of life in colorectal cancer survivors: A double-blind, randomized, placebo-controlled trial	Not on patients with bowel dysfunction
Effects of Daikenchuto on postoperative gastrointestinal motility in colorectal carcinoma patients with abdominal pain and distension: a prospective, randomized trial	Not on patients with bowel dysfunction
Effects of Probiotics on Bowel Function Restoration following Ileostomy Closure in Rectal Cancer Patients: A Randomized Controlled Trial	Not on patients with bowel dysfunction
Effects of PrObiotics on the Symptoms and Surgical ouTComes after Anterior REsection of Colon Cancer (POSTCARE): A Randomized, Double-Blind, Placebo-Controlled Trial	Not on patients with bowel dysfunction
Efficacy of oral administration of cystine and theanine in colorectal	On chemotherapy toxicity

cancer patients undergoing capecitabine-based adjuvant chemotherapy after surgery: a multi-institutional, randomized, double-blinded, placebo-controlled, phase II trial (JORTC-CAM03)	
Functional Outcome, Quality of Life, and Efficacy of Probiotics in Postoperative Patients with Colorectal Cancer	Not on patients with bowel dysfunction
Implementation of Pelvic Floor Rehabilitation after rectal cancer surgery: A qualitative study guided by the Consolidated Framework for Implementation Research (CFIR)	Not on pharmacological treatments
Improvement Effect of Bifidobacterium animalis subsp. lactis MH-02 in Patients Receiving Resection of Colorectal Polyps: A Randomized, Double-Blind, Placebo-Controlled Trial	Not on patients with bowel dysfunction
Improvement of Diarrhea-Type Defecation Habit Changes and Other Complications After Dixon Surgery for Rectal Cancer With Discriminative Chinese Herbs Combined With Levator Ani Muscle Training	Not on stable patients with bowel dysfunction
Long-term survey of the treatment of diarrhoea with loperamide	Not on colorectal cancer surgery
Loperamide Versus Psyllium Fiber for Treatment of Fecal Incontinence: The Fecal Incontinence Prescription (Rx) Management (FIRM) Randomized Clinical Trial	Not on colorectal cancer surgery
Low anterior resection syndrome after rectal resection management: multicentre randomized clinical trial of transanal irrigation with a dedicated device (cone catheter) versus conservative bowel management	Not on pharmacological treatments
Pelvic Floor Rehabilitation After Rectal Cancer Surgery One-year follow-up of a Multicenter Randomized Clinical Trial (FORCE trial)	Not on pharmacological treatments
Personal experience with administration of Smektit (hydrated magnesium aluminum silicate) in the treatment of diarrhea of non-infectious origin and diarrhea after resection of the large intestine	No access to full-text
Postoperative Probiotics Administration Attenuates Gastrointestinal Complications and Gut Microbiota Dysbiosis Caused by Chemotherapy in Colorectal Cancer Patients	On chemotherapy toxicity
Randomised clinical trial: colestyramine vs. hydroxypropyl cellulose in patients with functional chronic watery diarrhoea	Not on colorectal cancer surgery
Randomized clinical trial of prophylactic transanal irrigation versus supportive therapy to prevent symptoms of low anterior resection syndrome after rectal resection	Not on patients with bowel dysfunction
Randomized clinical trial of effect of synbiotics, neomycin and mechanical bowel preparation on intestinal barrier function in patients undergoing colectomy	Not on patients with bowel dysfunction
Synbiotics and gastrointestinal function-related quality of life after elective colorectal cancer resection	Not on patients with bowel dysfunction
The effect of anti-adhesive agent on patients with low anterior resection syndrome	Retrospective design
The effect of perioperative probiotics treatment for colorectal cancer: short-term outcomes of a randomized controlled trial	Not on patients with bowel dysfunction
The effects of perioperative probiotic treatment on serum zonulin concentration and subsequent postoperative infectious complications after colorectal cancer surgery: a double-center and double-blind randomized clinical trial	Not on patients with bowel dysfunction
The impact of a modified microbiota-accessible carbohydrate diet on gut microbiome and clinical symptoms in colorectal cancer patients following surgical resection	Not on pharmacological treatments
The response of patients with bile acid diarrhoea to the farnesoid X receptor agonist obeticholic acid	Not on colorectal cancer surgery
Tricyclic antidepressants for the treatment of tenesmus associated with rectal prolapse	Not on colorectal cancer surgery

Usefulness of valproate sodium for treatment of incontinence after ileoanal anastomosis	No access to full-text
